# Morphological and proteomic study of waterlogging tolerance in cotton

**DOI:** 10.1038/s41598-024-64322-y

**Published:** 2024-06-24

**Authors:** Hao Zhang, Zhangshu Xie, Xiaoju Tu, Aiyu Liu, Jinxiang Chen, Yunxin He, Bibo Wu, Zhonghua Zhou

**Affiliations:** 1 Cotton Research Institute, College of Agronomy, Hunan Agricultural University, Changsha, 410128 China; 2 Key Laboratory of Ministry of Education for Crop Physiology and Molecular Biology, Changsha, 410128 China; 3 Hunan Institute of Cotton Science, Changde, 415101 China; 4 Hunan Biological and Electromechanical Polytechmic, Changsha, 410127 China

**Keywords:** Floating seedling cultivation technique, Aquatic root, Waterlogging tolerance, Morphology, Proteome, Biotechnology, Plant sciences

## Abstract

Floating seedling cultivation technique is a novel seedling method in cotton and it provides an ideal model to study cotton growing under waterlogging stress. Morphological character and proteomic profile of the primary root from the seedling cultured by the new technology were evaluated in this study. Compared to seedlings cultured by the traditional method, the diameter of the taproot from floating technology is small at all five seedling stages from one-leaf stage to five-leaf stage. There are similar changes between the thickness of cortex and diameter of stele, which increased from the one- to the two-leaf stage but decreased from the two- to the five-leaf stage. At the one-leaf stage, the number and volume of mitochondria in the primary root-tip cells were less than those in the control. At the two-leaf stage, there was significantly less electron-dense material in the primary root-tip cells than those in the control group. From the one- to the two-leaf stage, the vacuole volume was significantly smaller than that in the control. Total 28 differentially expressed proteins were revealed from aquatic and control group roots of cotton seedlings at the three-leaf stage by two-dimensional electrophoresis, which included 24 up-regulated and four down-regulated proteins. The relative expression of the phosphoglycerate kinase (*PGK*) gene in aquatic roots increased from the one- to the four-leaf stage but declined rapidly from the four- to the five-leaf stage. The relative expression of the *14–3-3b* gene tended to decrease from the one- to the five-leaf stage. The *PGK* and *14–3-3b* genes were specifically expressed in the aquatic roots at the three-leaf stage. In brief, these changes induced waterlogging resistance in the aquatic roots of cotton seedlings in the floating nursery, thereby causing the roots to adapt to the aquatic environment, promoting the growth and development of cotton seedlings.

## Introduction

In recent years, China's cotton production has undergone significant adjustments, with the planting area recovering year by year. However, the lack of labor and high planting costs have always been the main factors restricting the development of the production. Cotton light simplification aims to simplify the process of managing cotton planting, reduce the number of operations, and lower labour intensity. By integrating agro-mechanical and agro-technical practices and using high-quality seeds and techniques supported by a new technology system, cotton production can become more efficient, cost-effective, and increase economic benefits. In recent years, the cotton industry in China has faced labour shortages and increased labour costs. Light simplification of cotton could potentially spur the growth of the industry. Therefore, only by strengthening light simplification and mechanization is the main way out for the development of production.

Furthermore, in the cotton-growing regions of southern China, particularly in the middle and lower reaches of the Yangtze River, due to the region's geographical location and natural climate, cotton is frequently subjected to heavy rainfall during the sowing period. This coincides with the critical period for cotton seedling emergence and seedling growth and development. The heavy rainfall results in a peak in soil water content, which in turn causes serious waterlogging of the field. In the field, the cotton plant is flooded locally or as a whole, and the roots are subjected to the flooding stress, which results in the formation of flooding. This has a number of consequences for the physiological and biochemical characteristics and molecular mechanisms of cotton. The lack of oxygen in the underground part of the plant blocks the synthesis of dry matter in the above-ground part of the plant, which adversely affects the subsequent growth and development of cotton seedlings and their yields^1^. In conclusion, further analysis of the molecular mechanism of flooding tolerance in cotton is an important guarantee to promote the light simplification and mechanisation of cotton production and the stability of the cotton industry in the Yangtze River Basin in China.

Chen et al^2^ created a novel seedling culturing method named floating seedling cultivation technique. It is a new seedling technology that cultures cotton seed in multi-element nutrient solution. Compared with the traditional methods such as seedling cultivation in soil nursery pots, the new method has some distinguished advantages including reduced cost of labor, decreased hazard of pest and disease, higher seedling survival and shortened recovery period. The new seedling technology has been applied widely in most cotton belts in China due to its outstanding virtue. They found that seedling cultured by the new method could form an aquatic root system after a certain period of hypoxic induction in order to adapt to the aquatic environment. Floating nurseries provide cotton seedlings with a growth environment under waterlogging stress. The roots of cotton seedlings cultured by the new technology consist of two parts, aquatic roots and xeric roots. Aquatic roots grow in nutrient solution under a hypoxic environment, which may be responsible for water and nutrient absorption. However, xeric roots grow in the matrix and they may play roles in respiration for cotton seedlings.

Under flooded conditions, cotton plant height significantly decreases, and both lint and cotton seed yields decrease^3,4^. Wang et al^5^ found that under waterlogging stress, the content of soluble protein and MDA in cotton leaves significantly increased. The waterlogging treatment has a significant promoting effect on the morphological growth of cotton roots, but it only has a promoting effect more than 23 days after the waterlogging ends^6^.

Kong et al^6^ found that the root system diameter of hydroponically grown spider plant (*Chlorophytumcomosum*) was smaller than that of soil grown plants. The hydroponic roots contain much fewer crystalliferous cells and amyloplasts in the root cap compared to xeric plants, fewer cortical cells and crystalliferous cells in the mature root regions, and thicker endothelial layers and a smaller pith area in the stele. However, the hydroponic roots can’t form complete aerenchyma tissue and the cell gap is relatively small and irregular. Zhen et al^8^ found that waterlogging could lead to the formation of aerenchyma tissue at the tillering stage in rice. The mechanism of cotton to adapt to waterlogging stress is to accelerate growth, form adventitious root and produce aerenchyma^9^.

Waterlogging can have a significant impact on the expression of genes related to photosynthesis, reactive oxygen species (ROS) clearance, anaerobic metabolism, and cell growth in cotton leaves. This includes *LHCB*, *CSD*, *ACS6*, *ADH*, *PDC*, *ERFs*, *XTHs*, and *EXPAs*, which may play a role in adapting to waterlogging stress^10^. Zhang et al^11^ found that 794 genes were up-regulated and 1018 genes were down-regulated in cotton leaves under waterlogging treatment, and these genes were associated with photosynthesis, nitrogen metabolism, starch and sucrose metabolism, glycolysis and hormone signalling, flavonoid synthesis, oxidative phosphorylation, amino acid metabolism and synthesis, biological clock, and other pathways. Wang et al^12^ compared the protein expression of aquatic roots and xeric roots in cotton, and identified 169 differentially expressed proteins, including 116 up-regulated proteins and 53 down-regulated proteins. Christianson et al^13^ used a gene chip to assess the growth response of cotton to waterlogging and found that flooding altered the expression of 1012 genes involved in biological processes such as glycolysis and anaerobic fermentation in root tissue as early as 4 h after flooding.

Most abiotic stresses including drought and salt have been studied by more and more researchers in cotton, but the molecular mechanisms on flood resistance have not been widely reported. The team in the previous study, through the use of 2-D gel electrophoresis has been detected floating seedlings of cotton aquatic roots and the control group of different proteins, and then on the basis of this study, the morphological and proteomic profile of aquatic roots cultured by floating seedling method, which simulates waterlogging stress was investigated, and xeric roots cultured by the traditional method was regarded as control. The results of this study could provide a reference for waterlogging resistance in cotton seedlings, and may also provide candidate genes for molecular breeding of cotton or other crops for flooding resistance within a certain range.

## Materials and methods

### Plant material

The upland cotton variety Zhongmiansuo 12 (waterlogging resistant cotton varieties)^14^, a widely planting cultivar was used in this study, which was provided by the National Medium-term Gene Bank of Cotton in China, Cotton Research Institute, Chinese Academy of Agricultural Sciences

### Cultivation of seedlings

**Figure 1:**
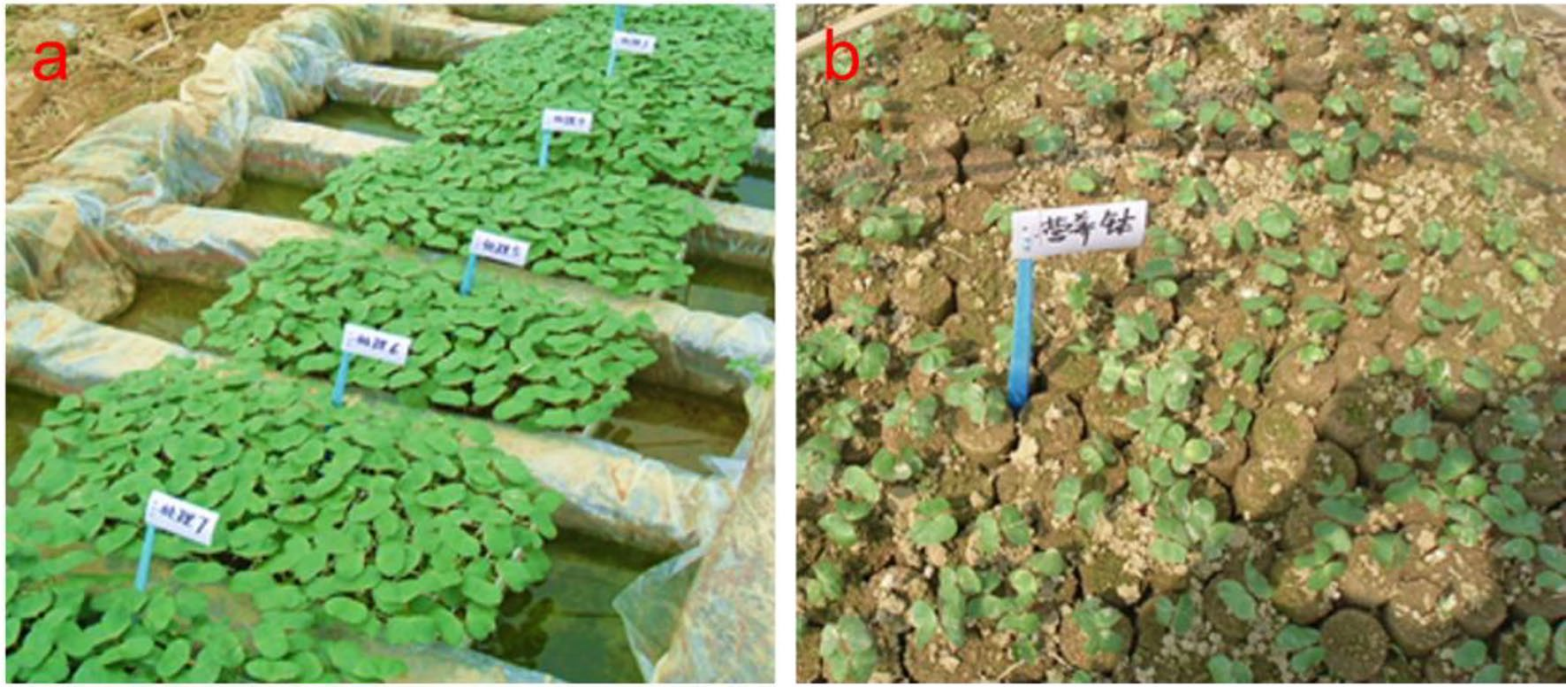
Cotton seedlings in the floating nursery with control. Note: (**a**) cotton floating seedlings; (**b**) cotton conventional method seedlings.

The seedling test was conducted at the Institute of Cotton Research of Hunan Agricultural University and the State Key Laboratory of Tree Genetics and Breeding, Chinese Academy of Forestry, Beijing, China from September 2011 to August 2013 (Fig. 1).

In the test group, the floating nursery was prepared as proposed by Chen et al.^2^. First, the cotton seeds were germinated, and the substrate was then mixed with an appropriate amount of water and used to fill the seedling tray. Cotton seeds were then sown in the seedling tray and covered with the substrate. After the cotyledons were unearthed, the seedling trays were placed in a plastic pot containing nutrient solution in an artificial climate greenhouse, while the nutrient solution was changed once every other week (This process prevents the growth of algae, such as green algae, on the aquatic roots of young cotton seedlings, which can otherwise cause the seedlings to die.). A substrate nursery was established as a control group, in which cotton seeds were first germinated and then the substrate was homogeneously mixed with an appropriate amount of water. Then, the mixture was used to fill aplastic cup, and cotton seeds were sowed in a plastic cup under coverage of the substrate. Next, each cup was watered with 20–30 mL of nutrient solution at the same concentration as the floating nursery. The artificial climate greenhouse was set to 28 °C, and daylight illumination was provided for 13 h with light intensity of 24,000 lx. The substrate used in the floating and substrate nursery, the nursery fertilizer, and the seedling tray were developed by Hunan Agricultural University and produced and supplied by Hunan Shui mu Biotechnology Co., Ltd. (Substrate composition was vermiculite powder: grass charcoal: expanded perlite: grass ash: plant straw = 3:4:1:1:1 (v/v). Nutrient solution formula: pure nitrogen concentration of 247.3–300 mg/L, effective phosphorus concentration at 60-151.2 mg/L, effective potassium concentration at 60–140 mg/L, iron 16–30 mg/L, boron 0.52–2.64 mg/L, manganese 0.7–4.4 mg/L, zinc 0.72 2.92 mg/L, copper 0.44–0.89 mg/L.)

### Morphological analysis of the primary roots of cotton seedlings in the floating nursery

The State Key Laboratory of Tree Genetics and Breeding, Chinese Academy of Forestry, Beijing, China from September 2011 to August 2013.

Samples were collected from the one- to five-leaf stage, and 10 seedlings at the same level of development as the test group were randomly selected. The experiment was run in triplicate. First, cotton seedlings were carefully lifted off the floating plate or substrate and rinsed with clean water to remove impurities while limiting the loss of roots. A 20 mm section of the primary root tip was cut and fixed with FAA fixative solution (90 mL of 70 %ethanol, 5 mL of glacial acetic acid, and 5 mL of formaldehyde) for 1–2 days, and then a permanent film was prepared as previously described^15,16^, and subsequently observed using a Carl Zeiss Axio Imager A1 optical microscope. Three slices were prepared for each root sample. Five sections were selected on each slice as sample points for observation and imaging. The diameter, cortical thickness, and stele diameter of each main tip were measured and averaged, respectively.

### Observation of the primary root tip ultrastructure of cotton seedlings in a floating nursery

Root tips (2–3 mm long) were collected from the one- to five-leaf stage of the control and test groups, and separately fixed in penicillin vials containing 3% glutaraldehyde; the procedure was performed in triplicate. After treatment at room temperature for 1–2 days, ultra-thin sections were prepared as described by Li et al.^17^, and then double-stained with uranyl acetate and lead citrate. Samples were then observed and the ultrastructure of the root tips was imaged under a Hitachi H-7500 transmission electron microscope (TEM).

### Bidirectional protein electrophoresis and proteomic analysis of the aquatic roots of cotton seed-lings in a floating nursery

The aquatic roots of the control and test groups were cut at the three-leaf stage, snap-frozen in liquid nitrogen, and stored in a refrigerator at -80 °C, with the whole procedure performed in triplicate. Total proteins were extracted from cotton roots and quantified, and then subjected to two-dimensional protein electrophoresis, gel staining, gel mapping analysis, differential protein spot analysis with mass spectrometry, and bioinformatics analysis^18,19^. Samples were analyzed by 4700 MALDI-TOF/TOF Proteomics Analyzer (Applied Biosystems, Foster City, CA). GPS Explorer software (Applied Biosystems, Foster City, CA; Version 3.0) was used to create and search files in the MASCOT search engine (version 2.0; Matrix Science, London, U.K.) for peptide and protein identification with the following parameters: NCBInr database or Swiss-Prot database.

### Analysis of key gene expression in the roots of seedlings in a floating nursery

Aquatic roots were cut from the one- to five-leaf stage of the control and test groups, and young stems and leaves were cut from each group at the 3-leaf stage. All samples were snap-frozen in liquid nitrogen and stored in a refrigerator at − 80 °C, with all procedures performed in triplicate. Total RNA was extracted from the above materials, purified, and reverse-transcribed into cDNA. Primer 3.0 software was used to design specific primers for the two key genes involved in glycolysis and the starch synthesis pathway, as follows: phosphoglycerate kinase (p1: GCCTTCTGTTGCTGGTTTTCTTp2: CCACAAGTGAAGATCCAACTGC), and 14-3-3bI1: CCTCTCCGTCGCTTACAAGAAT I2: AGGGGACAAGCCTAGTGTCAAG), with the Histone3 gene acting as an internal reference to design the specific primers (h1: TCAAGACTGATTTGCGTTTCCA; h2: GCGCAAAGGTTGGTGTCTC).The above cDNAs were used as a templates for real-time quantitative fluorescence PCR^20^. Data were analyzed using SPSS software.

### Guideline

Experimental research and field studies on plants (either cultivated or wild), including the collection of plant material, comply with relevant institutional, national, and international guidelines and legislation.

## Results and analysis

### Morphological comparison of the primary roots of cotton seedlings in the floating nursery

#### Comparison of the primary root tip diameter of cotton seedlings in the floating nursery

**Figure 2:**
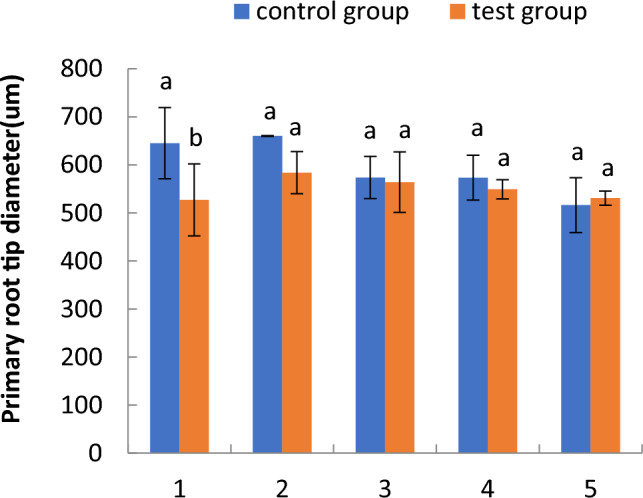
Comparison of primary root tip diameter of cotton seedling in the floating nursery. Note: (**1**) one-leaf and one-heart stage; (**2**) two-leaf and one-heart stage; (**3**) three-leaf and one-heart stage; (**4**) four-leaf and one-heart stage; (**5**) five-leaf and one-heart stage. Different letters indicate significant differences (*p* < 0.05) between treatments and controls according to a Duncan’s test. Error bars are all standard deviations. The same as below.

Figure 2 shows the variation in the primary root tip diameter of cotton seedlings in a floating nursery. The primary root tip diameter of seedlings in the floating nursery was significantly smaller than that of the control group in the one-leaf stage, smaller in the two- to four-leaf stage, but slightly larger than that of the control group in the five-leaf stage. The primary root tip diameters of seedlings in the floating nursery tended to increase from the one- to two-leaf stage but gradually decreased from the two- to the five-leaf stage.

#### Cortical thickness of primary root tips of cotton seedlings in the floating nursery

**Figure 3:**
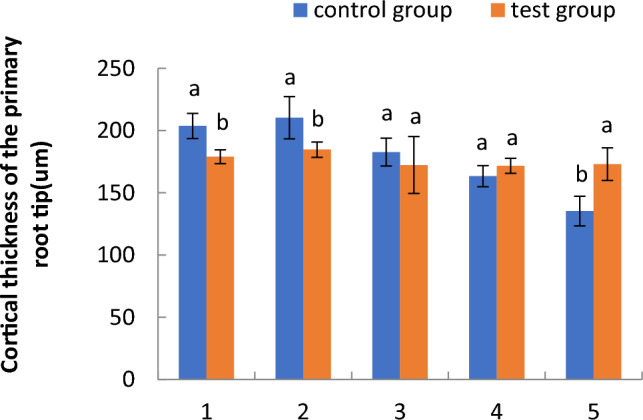
Comparison of the cortical thickness of primary root tips of cotton seedlings in the floating nursery. Note: (**1**) one-leaf and one-heart stage; (**2**) two-leaf and one-heart stage; (**3**) three-leaf and one-heart stage; (**4**) four-leaf and one-heart stage; (**5**) five-leaf and one-heart stage.

As shown in Fig. 3, the cortical thickness of primary root tips of cotton seedlings in the floating nursery was significantly less than that of the control group in the one- and two-leaf stages, and slightly less in the three-leaf stage. However, it was slightly larger than that of the control group in the four-leaf stage and significantly larger than in the five-leaf stage. The cortical thickness of primary root tips from seedlings in the floating nursery tended to increase slowly from the one- to the two-leaf stage and then slowly decrease from the two- to the five-leaf stage.

#### Comparison of the stele diameter of primary root tips of cotton seedlings in the floating nursery

**Figure 4:**
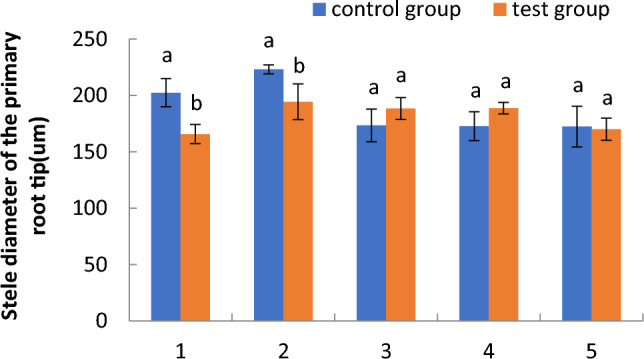
Comparison of the stele diameter of primary root tips of cotton seedlings in the floating nursery. Note: (**1**) one-leaf and one-heart stage; (**2**) two-leaf and one-heart stage; (**3**) three-leaf and one-heart stage; (**4**) four-leaf and one-heart stage; (**5**) five-leaf and one-heart stage.

Variation in the stele diameter of primary root tips of cotton seedlings in the floating nursery is shown in Fig. 4. The stele diameter of primary root tips of cotton seedlings in the floating nursery was significantly smaller than that of the control group from the one- to the two-leaf stage, and slightly larger than that from the three- to the four-leaf stage, but very similar to that from the control group in the five-leaf stage. The stele diameter of primary root tips of seedlings in the floating nursery tended to increase from the one- to the two-leaf stage and slowly decreased from the two- to the five-leaf stage.

### Comparison of the primary root tip ultrastructure of cotton seedlings in the floating nursery

**Figure 5:**
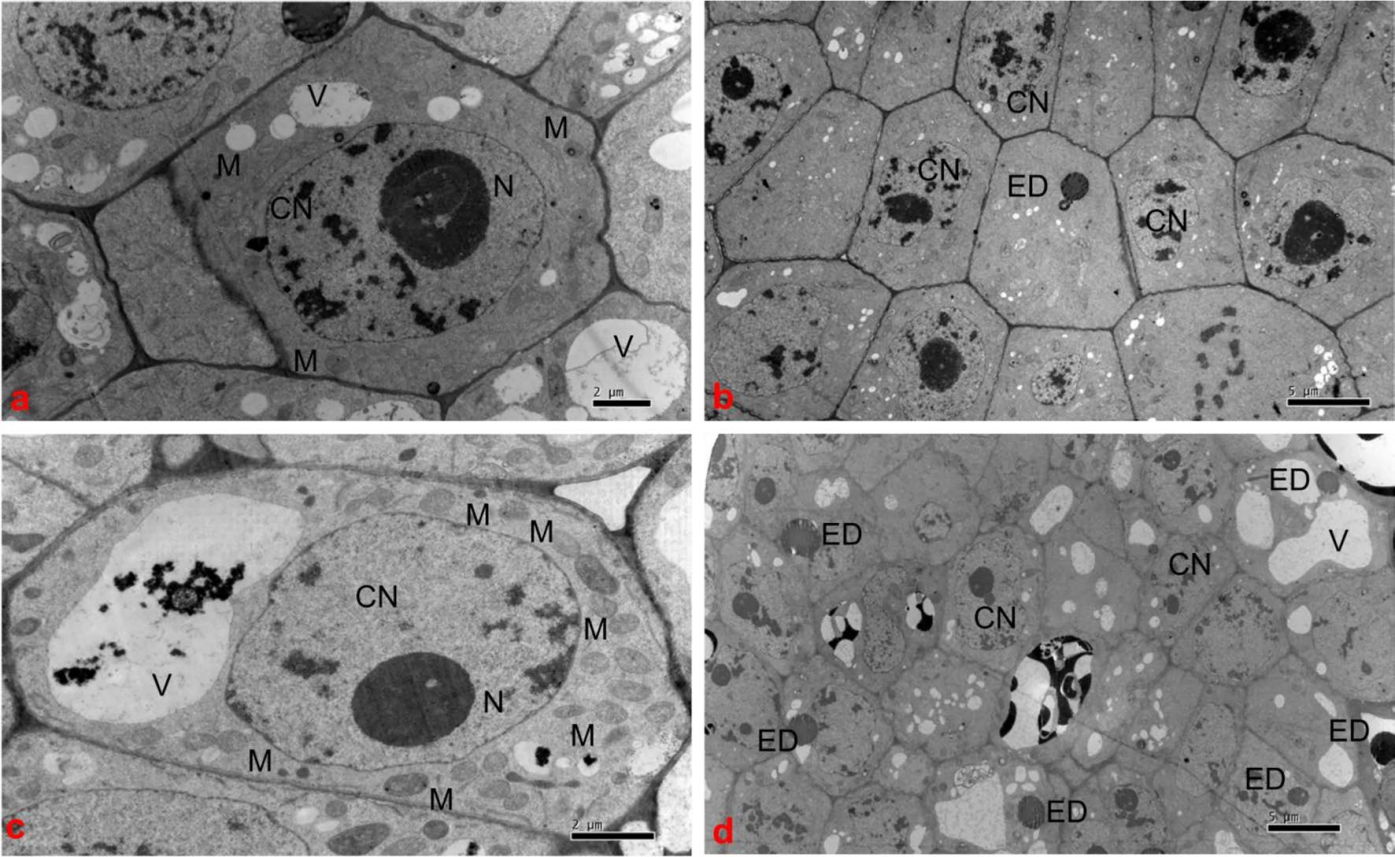
Ultrastructure of the primary root tips of cotton seedlings in the floating nursery. Note: (**CN**): cell nucleus; (**N**): nucleolus; (**V**): vacuole; (**M**): mitochondria; (**ED**): electron-dense material. The experimental group includes one-leaf and one-heart stage floating nursery cotton seedling primary root tip cells (**a**) and two-leaf and one-heart stage floating nursery cotton seedling primary root tip cells (**b**). The control group includes one-leaf and one-heart stage basal nursery cotton seedling primary root tip cells (**c**) and two-leaf and one-heart stage basal nursery cotton seedling primary root tip cells (**d**).

As shown in Fig. 5a, there were significantly fewer mitochondria in the primary root tip cells of cotton seedlings in the floating nursery than in those of the control group (Fig. 5c). Furthermore, the volume of mitochondria was lower than in the control group, and the vacuole volume of primary root tip cells was significantly lower compared with the control group. As shown in Fig. 5b, there were significantly fewer electron-dense substances (lysosomes) in the primary root tip cells of cotton seedlings in the floating nursery than in those of the control group at the two-leaf stage (Fig. 5d), and the vacuole volume was also significantly smaller than that in the control group.

### Two-dimensional protein electrophoresis analysis of the aquatic roots of cotton seedlings in a floating nursery

For the three-leaf stage, two-dimensional protein electrophoresis of aquatic roots in the floating nursery and control roots revealed 49 reproducible differential protein spots, which were reduced to 28 with MALDI TOF/TOF MS and a database search. These consisted of 24 up-regulated proteins and four down-regulated proteins (Table 1).

**Table 1:** Proteins expressed in the aquatic roots of cotton seedlings in the floating nursery.

No	Accession number	Protein name	Protein function	Classification	Expression
1	gi|354,620,275	VdI2 protection protein	Cell defense	Defense	UP
2	gi|89,276,303	Actin-depolymerizing factor 6	Cytoskeleton	Cell structure	UP
3	gi|89,276,297	Actin-depolymerizing factor 3	Cytoskeleton	Cell structure	UP
4	gi|295,687,227	Eukaryotic translation initiation factor5A	Positive regulation of translational elongation	Protein synthesis	UP
5	gi|34,421,682	Katanin-like protein	Cytoskeleton	Cell structure	UP
6	gi|354,620,271	MLP protection protein	Cell defense	Defense	UP
7	gi|283,488,489	UDP-glucose dehydrogenase	Oxidation–reduction	Metabolism	Down
8	gi|1,263,291	Alcohol dehydrogenase 2b	Alcoholic fermentation	Metabolism	UP
9	gi|193,290,377	14–3-3b protein	Starch metabolism	Metabolism	Down
10	gi|56,475,246	Unknown protein	Unknown function	Unknown	UP
11	gi|211,906,490	malate dehydrogenase	TCA	metabolism	UP
12	gi|22,531,587	MYB153 protein	Cell division	Cell growth	Down
13	gi|310,787,928	14–3-3-like protein	Starch metabolism	Metabolism	Down
14	gi|211,906,490	Malate dehydrogenase	TCA	Metabolism	UP
15	gi|1,220,196	Alcohol dehydrogenase2a	Alcoholic fermentation	Metabolism	UP
16	gi|1,220,196	Alcohol dehydrogenase2a	Alcoholic fermentation	Metabolism	UP
17	gi|211,906,446	Adenosine kinase	Biological synthesis of adenosine monophosphate	Metabolism	UP
18	gi|1,220,196	Alcohol dehydrogenase2a	Alcoholic fermentation	Metabolism	UP
19	gi|211,906,450	Phosphoglycerate kinase	Glycolysis	Metabolism	UP
20	gi|1,263,291	Alcohol dehydrogenase 2b	Alcoholic fermentation	Metabolism	UP
21	gi|211,906,450	Phosphoglycerate kinase	Glycolysis	Metabolism	UP
22	gi|1,263,291	Alcohol dehydrogenase 2b	Alcoholic fermentation	Metabolism	UP
23	gi|211,906,502	Isocitrate dehydrogenase	TCA	Metabolism	UP
24	gi|158,144,895	Enolase	Glycolysis	Metabolism	UP
25	gi|158,144,895	Enolase	Glycolysis	Metabolism	UP
26	gi|158,144,895	Enolase	Glycolysis	Metabolism	UP
27	gi|211,906,444	Fructokinase	Glycolysis	Metabolism	UP
28	gi|242,129,046	F1-ATPase β-subunit	Biological synthesis of adenosine triphosphate	Energy	UP

### Expression analysis of key flood resistance genes in the aquatic roots of cotton seedlings in the floating nursery

#### Expression of the phosphoglycerate kinase gene

**Figure 6:**
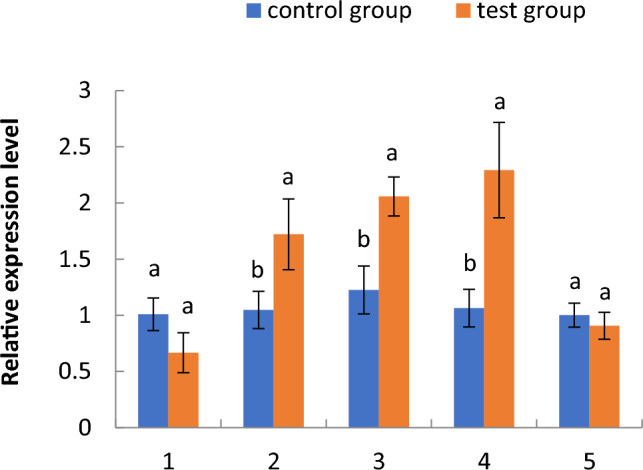
Relative expression of the phosphoglycerate kinase (PGK) gene in the aquatic roots of cotton seedlings in the floating nursery at different seedling stages. Note: (**1**) one-leaf and one-heart stage; (**2**) two-leaf and one-heart stage; (**3**) three-leaf and one-heart stage; (**4**) four-leaf and one-heart stage; (**5**) five-leaf and one-heart stage.

**Figure 7:**
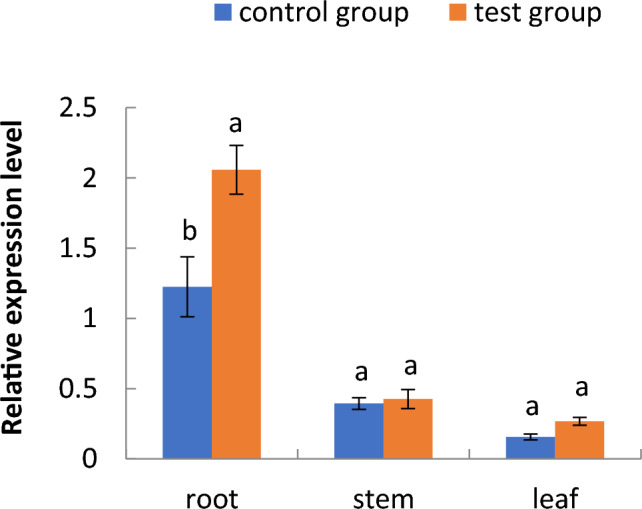
Relative expression of the phosphoglycerate kinase gene in different parts of cotton seedlings in the floating nursery.

As shown in Fig. 6, the relative expression of the phosphoglycerate kinase gene in the aquatic roots of cotton seedlings in the floating nursery at the one-leaf stage was lower than that of the control group, significantly higher from the two- to the four-leaf stage, and lower again in the five-leaf stage. The relative expression of this gene in aquatic roots tended to increase from the one- to the four-leaf stage, but decreased sharply from the four- to the five-leaf stage. As shown in Fig. 7, the relative expression of this gene in aquatic roots of three-leaf stage seedlings in the floating nursery was significantly higher than in the control group, while the relative gene expression in the stems and leaves was not significantly different from the control group.

#### Expression of the 14–3-3b gene

**Figure 8:**
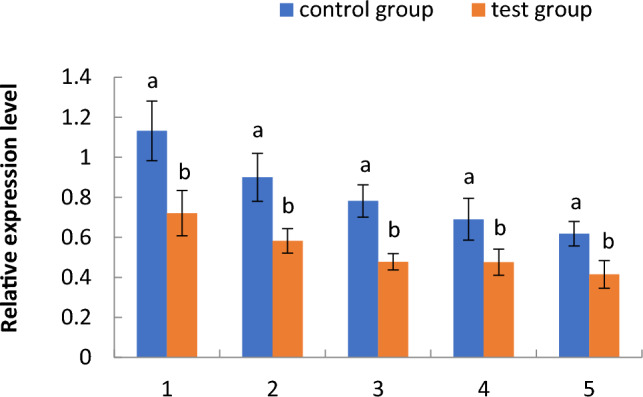
Relative expression of the 14–3-3b gene of aquatic roots of cotton seedlings in different seedling stages in the floating nursery. Note: (**1**) one-leaf and one-heart stage; (**2**) two-leaf and one-heart stage; (**3**) three-leaf and one-heart stage; (**4**) four-leaf and one-heart stage; (**5**) five-leaf and one-heart stage.

**Figure 9:**
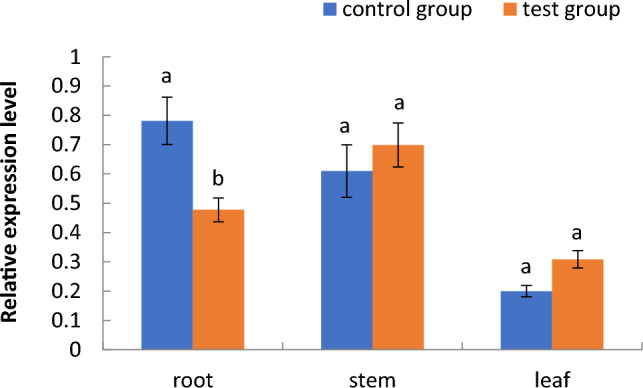
Relative expression of the 14–3-3b gene in different parts of cotton seedlings in the floating nursery.

As shown in Fig. 8, the relative expression of the 14-3-3b gene in the aquatic roots of cotton seedlings in the floating nursery was significantly lower than that in the control group from the one- to the five-leaf stage and showed a decreasing trend. As shown in Fig. 9, the expression of the 14-3-3b gene in seedlings at the three-leaf stage was significantly lower in the floating nursery compared with the control group; however, there was no significant difference in the expression of this gene in the stems and leaves of the test and control groups.

## Discussion

### Morphological analysis of the primary roots of cotton seedlings in the floating nursery

The diameter of the primary root tip of aquatic roots of cotton seedlings in the floating nursery in the one-leaf stage was significantly smaller than that of the control group. It is possible that the aquatic environment affects the growth and development of aquatic roots, which may in turn influence the survival of floating cotton seedlings after transplantation. However, it is also evident that seedlings that have undergone training and acclimatisation to the aquatic environment are more likely to survive after transplantation. Over time, the aquatic roots of cotton seedlings in the floating nursery grew from the one- to the two-leaf stage, and the diameter of the primary root tip slowly increased. From the two- to the five-leaf stage, the rate of aquatic root growth for cotton seedlings in the floating nursery slowed, and the diameter of the primary root tip gradually decreased. Regarding cortical thickness, the diameter of the primary root tipoff cotton seedlings in the floating nursery slowly increased from the one- to the two-leaf stage and slowly decreased from the two- to the five-leaf stage. The overall change from the one- to the five-leaf stage tended to be relatively stable and flat, indicating that the cortical thickness of the primary root tip of aquatic roots did not change significantly with the change of primary root tip diameter across the five stages. Cortical tissue is an important component of the lateral transport pathway of primary roots. Its growth was relatively stable, probably because the primary roots were in a highly hydrated environment, and they were able to actively absorb water in a stable manner, leading to no significant change in the thickness of cortical tissue. The stele diameter of the primary root tip changed in a manner consistent with the primary root tip diameter, which was also consistent with the growth state of the root system.

### Analysis of the primary root tip ultrastructure of cotton seedlings in the floating nursery

The main function of mitochondria is to supply energy to cells. Under waterlogging stress, the nuclei and mitochondria in the mid-segment cells of the main root of tolerant peanut varieties are basically normal^21^. In the one-leaf stage, there were significantly fewer mitochondria in cells of the primary root tip of cotton seedlings in the floating nursery than in those of the control group. This was probably because in the floating nursery environment, the loss of aquatic root cells from cotton seedlings was reduced, and cells divided less than in the control group. In a comprehensive analysis, the root system of cotton seedlings growing in the soil in the field must rub against soil particles in order to elongate and expand, and therefore must lose a large number of root cells. In contrast, floating seedling root systems do not need to rub against soil particles, and therefore, the amount of root cell loss is very small, with a corresponding reduction in the degree of cell division. Furthermore, analysis of the ultrastructural images revealed a significant reduction in the number of mitochondria in the main root tip cells of the floating nursery cotton seedlings compared to the control. This suggests that the energy requirements of the root system of the floating cotton seedlings were also reduced, indicating a reduction in cell division in the main root tip cells of the floating cotton seedlings. The main function of lysosomes is to participate in normal digestion in cells. After entering the cell by endocytosis, macromolecular substances are digested by lysosomes and decomposed into small molecules, which then diffuse into the cytoplasm to nourish the cell. In the two-leaf stage, the number of lysosomes in the primary root tip cells of cotton seedlings in the floating nursery was significantly lower than in the control group. This may be because in the floating nursery environment, the loss of aquatic root cells from cotton seedlings was reduced, and the rate of cell division was also lower than in the control group, leading to a reduced requirement for nutrients by aquatic roots, and thus, fewer lysosomes in aquatic root cells compared with the control group. Vacuoles are highly permeable, allowing water to diffuse into the vacuole, which plays an important role in regulating osmotic pressure and maintaining turgor pressure in the cell. From the one- to the two-leaf stage, the vacuole volume of the primary root tip cells of cotton seedlings in the floating nursery was significantly lower than that of the control group. This was probably because in a highly hydrated environment, the ability of the primary roots to absorb water was relatively stable, so the vacuoles used to store water grew relatively slowly, and their volume was significantly less compared with the control group.

### Differential protein analysis in the aquatic roots of cotton seedlings in the floating nursery

#### Functional analysis of differential metabolic proteins

The main role of fructokinase (FRK) is to regulate glycolysis, sucrose, and starch conversion^11–23^. FRK was upregulated in the aquatic roots of cotton seedlings in the floating nursery for two reasons: (1) to improve the efficiency of the glycolytic pathway and more fully utilize sucrose to provide energy needed for the growth of aquatic roots; and (2) to promote the conversion of residual sucrose to starch in order to increase starch granules in the aquatic roots of cotton seedlings in the floating nursery, which was consistent with previous TEM observations made by our research group^24^. This indicates that in the floating nursery environment, the aquatic roots of cotton seedlings stored energy in the form of starch granules for root growth.

Phosphoglycerate kinase (PGK) is a key enzyme involved in the glycolytic pathway. Mutated or loss of PGK seriously impairs normal physiological functioning and can even lead to death^25^. Two proteins were upregulated in the aquatic roots of seedlings at the three-leaf stage in the floating nursery, which may promote the efficiency of the glycolytic pathway.

Alcohol dehydrogenase (ADH) is an important hydrolase involved in plant ethanol fermentation. ADH is one of the most highly upregulated enzymes in plants under a hypoxic environment induced by waterlogging^26^. As the aquatic roots of cotton seedlings in the floating nursery were in an anoxic environment, thee expression of ADH-2a and ADH-2b in these roots was up-regulated. This study found that Alcohol dehydrogenase (ADH) is a differentially expressed protein in the aquatic roots of floating cotton seedlings, which is consistent with the results of the study by Wang et al^12^.

Isocitrate dehydrogenase (ICDH) is a rate-limiting enzyme of the tricarboxylic acid (TCA) cycle. High levels of NADPH synthesized by ICDH can maintain the antioxidant system of plant cells to resist oxidative stress caused by the accumulation of reactive oxygen species (ROS)^27,28^. ICDH expression was upregulated in the aquatic roots of cotton seedlings in the floating nursery, which may be explained by two reasons. First, with the expansion of aerenchyma in the aquatic roots, the amount of oxygen transported from the shoots to the aquatic roots gradually increased; thus, there was an increase in aerobic respiration, which had been inhibited by hypoxia, namely the TCA pathway. Second, in the hypoxic environment, free radicals such as ROS were produced in the aquatic roots of cotton seedlings in the floating nursery, whereas ICDH may be involved in the degradation of free radicals, thereby reducing their effect on cell growth.

Malate dehydrogenase (MDH) is a key enzyme involved in sugar metabolism. In eukaryotic cells, mitochondrial MDH (mtMDH) is a key enzyme that provides energy in the TCA cycle. MDH was upregulated in the aquatic roots of cotton seedlings in the floating nursery, possibly promoting the efficiency of the TCA cycle.

14-3-3 is a small-molecule regulatory protein^29^. Starch synthesis in plants is counter-regulated by 14-3-3. Sehnke et al.^30^ found that inhibiting the activity of 14-3-3 in Arabidopsis leaves induced the accumulation of starch in leaves. This protein was down regulated in the aquatic roots of cotton seedlings in the floating nursery, and the amount of starch granules in the roots increased correspondingly, which was consistent with previous TEM observations made by our research group^24^.

UDP-glucose dehydrogenase (UDPGDH) is a key enzyme involved in polysaccharide synthesis^31^. In higher plants, UDPGDH is involved in hemicellulose synthesis and is closely related to the formation of plant cell walls^32^. UDPGDH was down regulated in the aquatic roots of cotton seedlings in the floating nursery. This was likely because in the aquatic environment, growth resistance of root tips was low and the degree of damage to root tip cells was also reduced. Thus, the main role of the cell wall is to protect cells, and this was correspondingly weakened, leading to down regulation of UDPGDH expression.

Adenosine kinase (ADK), a housekeeping gene^33^, was up-regulated in aquatic roots of three-leaf stage seedlings in the floating nursery. This was likely because ADK regulated cyclic adenosine monophosphate (cAMP) content, thereby regulating the expression of related genes for adaptation to the aquatic environment.

#### Functional analysis of differential cell structure proteins

Actin-depolymerizing factor (ADF) depolymerizes actin^34^. Actin forms the microfilament system of the cytoskeleton, which is involved in regulating the transport of starch granules in the root tip cells of plants in response to gravity, thereby achieving gravitropic (downward) growth. In the aquatic environment of seedlings in the floating nursery, the aquatic roots of cotton seedlings were suspended in water, and the characteristics of downward growth were weakened. This may be because the expression of ADF was up-regulated and actin was excessively depolymerized, resulting in an abnormal microfilament system.

Katanin is a microtubule-binding protein^35,36^. A katanin-like protein was previously identified in Arabidopsis mutants, and was found to regulate the normal orientation of microtubules and thus maintain normal cellular morphology^37^. Katanin was up regulated in the aquatic roots of cotton seedlings in the floating nursery, possibly because in the aquatic environment, this protein was required to maintain the normal morphology of root cells.

#### Functional analysis of differential cell defense proteins

VdI2 and MLP were both up regulated in the aquatic roots of cotton seedlings in the floating nursery, probably because the two proteins acted to protect root cells in the aquatic environment.

#### Functional analysis of differential energy metabolism protein (F1-ATPase β-subunit)

Adenosine triphosphate (ATP) synthase (ATPase) is the core enzyme involved in energy conversion in organisms, which synthesizes ATP in a manner dependent on the transmembrane proton potential^38^. The ATPase β-subunit is involved in energy metabolism^39^, and was up-regulated in aquatic roots of the three-leaf stage seedlings in the floating nursery, possible to catalyze the synthesis of ATP to provide the energy needed for aquatic root growth.

#### Functional analysis of differential protein-synthesizing proteins (eukaryotic translation initiation factor 5A)

Eukaryotic translation initiation factor 5A (eIF5A) is a conserved protein with a low-molecular weight^40^. In Arabidopsis, eIF5A promotes cell division, thereby promoting plant growth and development^41^. eIF5A was up regulated in the aquatic roots of cotton seedlings in the floating nursery, likely to promote the division of root cells and increase the number of cells in the root tip tissues.

#### Functional analysis of differential cell growth proteins (MYB153)

MYB is a transcription factor involved in the formation and elongation of root hair^42^. In the aquatic environment, root hair growth is inhibited and even degraded in spider plants^7^. At the three-leaf stage, MYB153 was downregulated in the aquatic roots of cotton seedlings in the floating nursery, possibly inhibiting root hair growth.

### Analysis of key flood resistance genes in the aquatic roots of cotton seedlings in the floating nursery

#### Expression of the PGK gene in the aquatic roots of cotton seedlings in the floating nursery

Aquatic roots of cotton seedlings in the floating nursery were exposed to a hypoxic environment. In the initial breeding stage of the floating nursery, namely the one-leaf stage, the cotyledons of cotton seedlings also provide energy for the growth of aquatic roots. Therefore, expression of PGK in aquatic roots was not significantly different from that in the control group. Over time, from the two- to the four-leaf stage, the leaf could no longer provide sufficient energy for the aquatic roots, and the glycolytic pathway involving the PGK gene was gradually strengthened. Thus, the expression of PGK was significantly higher than in the control group from the two- to the four-leaf stage, increased from the one- to the four-leaf stage, and peaked at the four-leaf stage. The oxygen supply gradually increased from the four- to the five-leaf stage with aerenchyma expansion in the roots, and a gradual weakening of the glycolytic pathway. This resulted in a sharp decrease in the relative expression of this gene in aquatic roots during this period. The relative expression of PGK in aquatic roots of cotton seedlings in the floating nursery was significantly higher than that in the control group, while there was no significant difference in the stems and leaves between the test and control groups. This indicates that the gene is differentially expressed in the aquatic roots, and is therefore induced by the aquatic environment.

#### Expression of the 14–3-3b gene in aquatic roots of cotton seedlings in the floating nursery

In a floating nursery environment, sucrose generated by photosynthesis is transported to the roots to provide energy or root growth, and the remaining sucrose is converted to starch for storage. The function of 14-3-3 is to counter-regulate the synthesis of starch in plants. The relative expression of the 14-3-3b gene in aquatic roots of cotton seedlings in the floating nursery was significantly lower than that in the control group from the one- to the five-leaf stage, indicating that the activity of 14-3-3b in the aquatic roots was inhibited, resulting in starch accumulation. Moreover, the relative expression of this gene in aquatic roots decreased from the one- to the five-leaf stage, leading to higher starch content in the aquatic roots than in the control group, which was consistent with previous TEM observations made by our research group. In the three-leaf stage, the relative expression of the 14-3-3b gene in aquatic roots of cotton seedlings in the floating nursery was significantly lower than that in the control group, while there was no significant difference in the relative expression in stems and leaves between the test and control groups. This indicates that the expression of this gene was induced by the aquatic environment (Supplementary Information file 1).

## Conclusions

In the floating nursery environment, the aquatic roots of cotton seedlings changed significantly in terms of primary root morphology, ultrastructure, proteome, and the expression of key genes, which stimulated flood resistance allowing the roots to adapt to the aquatic environment, there by promoting the growth and development of cotton seedlings.

## Research outlook and shortcomings

This article's results will serve as a valuable reference for identifying genes that confer flooding tolerance in cotton. The differential proteins obtained in this study are all related to cotton's flooding tolerance. Future work can involve expression analysis and functional verification of the gene corresponding to each protein to identify cotton flooding tolerance genes. The results only analysed the two genes in terms of gene expression and did not verify their gene function to determine whether they are flooding tolerance genes. This could be a potential area for future research.

### Supplementary Information

Supplementary Information 1.

Supplementary Information 2.

## Data Availability

The data that support the findings of this study are available from the corresponding author upon reasonable request.
